# Practical considerations for determination of scapular internal rotation and its relevance in reverse total shoulder arthroplasty planning

**DOI:** 10.1186/s13018-023-03762-0

**Published:** 2023-04-05

**Authors:** Paul Siegert, Dominik Meraner, Alexandra Pokorny-Olsen, Doruk Akgün, Gundobert Korn, Christian Albrecht, Jochen G. Hofstaetter, Philipp Moroder

**Affiliations:** 1 1st Orthopaedic Department, Orthopaedic Hospital Speising, Speisinger Str. 109, 1130 Vienna, Austria; 2 Michael Ogon Laboratory for Orthopaedic Research, Orthopaedic Hospital Speising, Vienna, Austria; 3 2nd Orthopaedic Department, Orthopaedic Hospital Speising, Vienna, Austria; 4 Department for Shoulder and Elbow Surgery, Charité - Universitätsmedizin Berlin, Berlin, Germany; 5 Department of Orthopaedic and Traumasurgery, Paracelsus Medical University, Salzburg, Austria; 6 Department of Shoulder and Elbow Surgery, Schulthess Clinic, Zurich, Switzerland

**Keywords:** Posture types, Scapulothoracic orientation, Reverse total shoulder arthroplasty, Scapular internal rotation

## Abstract

**Background:**

Scapulothoracic orientation, especially scapular internal rotation (SIR) may influence range of motion in reverse total shoulder arthroplasty (RTSA) and is subjected to body posture. Clinical measurements of SIR rely on apical bony landmarks, which depend on changes in scapulothoracic orientation, while radiographic measurements are often limited by the restricted field of view (FOV) in CT scans. Therefore, the goal of this study was (1) to determine whether the use of CT scans with a limited FOV to measure SIR is reliable and (2) if a clinical measurement could be a valuable alternative.

**Methods:**

This anatomical study analyzed the whole-body CT scans of 100 shoulders in 50 patients (32 male and 18 female) with a mean age of 61.2 ± 20.1 years (range 18; 91). (1) CT scans were rendered into 3D models and SIR was determined as previously described. Results were compared to measurements taken in 2D CT scans with a limited FOV. (2) Three apical bony landmarks were defined: (the angulus acromii (AA), the midpoint between the AA and the coracoid process tip (C) and the acromioclavicular (AC) joint. The scapular axis was determined connecting the trigonum scapulae with these landmarks and referenced to the glenoid center. The measurements were repeated with 0°, 10°, 20°, 30° and 40° anterior scapular tilt.

**Results:**

Mean SIR was 44.8° ± 5.9° and 45.6° ± 6.6° in the 3D and 2D model, respectively (*p* < 0.371). Mean difference between the measurements was 0.8° ± 2.5° with a maximum of 10.5°. Midpoint AA/C showed no significant difference to the scapular axis at 0° (*p* = 0.203) as did the AC-joint at 10° anterior scapular tilt (*p* = 0.949). All other points showed a significant difference from the scapular axis at all degrees of tilt.

**Conclusion:**

2D CT scans are reliable to determine SIR, even if the spine is not depicted. Clinical measurements using apical superficial scapula landmarks are a possible alternative; however, anterior tilt influenced by posture alters measured SIR.

## Introduction

**Fig. 1:**
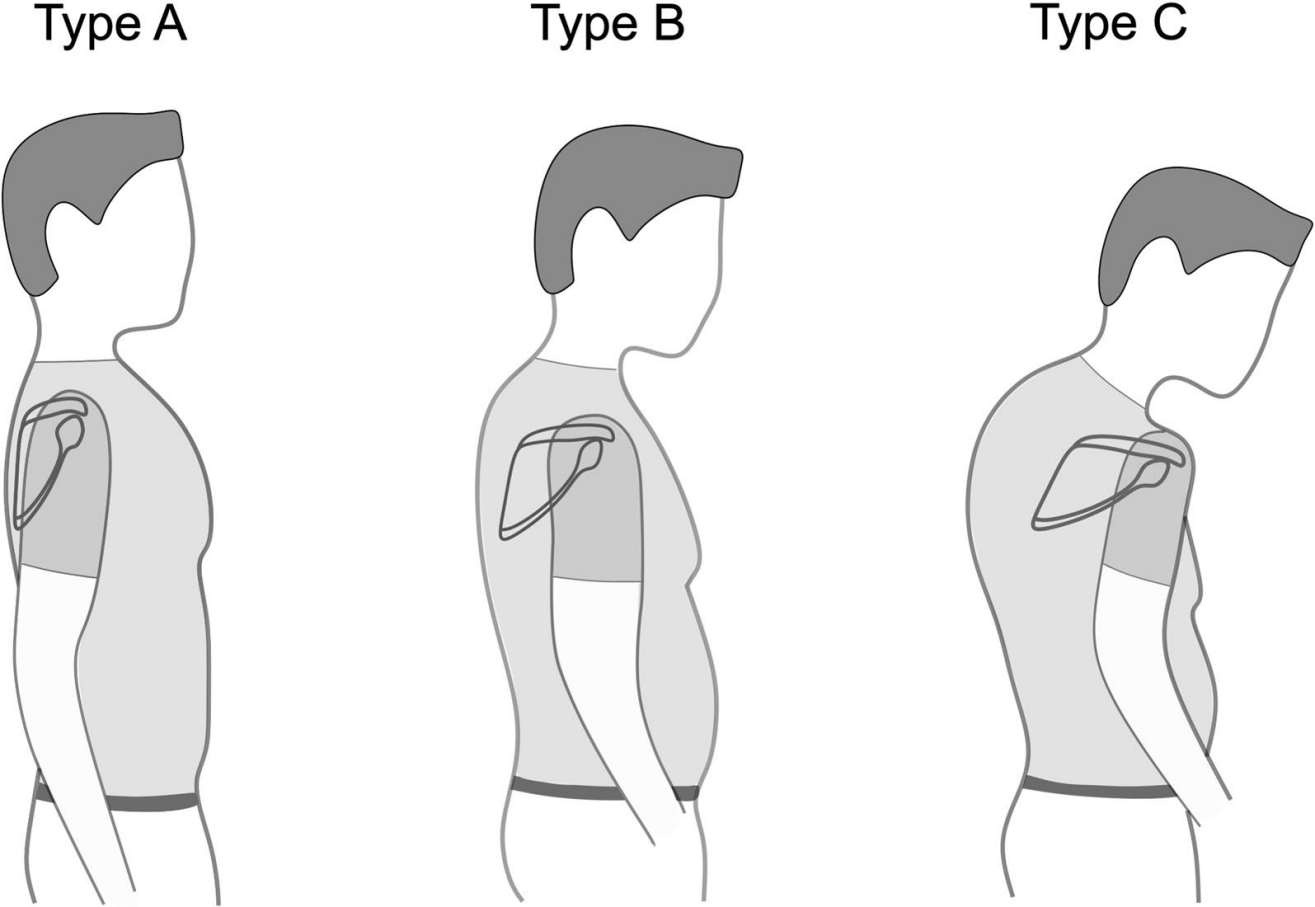
Illustration of three different posture types. From Type **A** over **B** to **C** patients show increasing scapular internal rotation, anterior tilt, protraction, and drooping as well as kyphosis and a barrel-shaped chest according to Moroder et al. [7]

Impaired internal and external rotation pose a challenge in reverse total shoulder arthroplasty (RTSA) [1–5]. The position of the scapula relative to the torso is defined by three motions: scapula internal/external rotation, anterior/posterior tilt and upward/downward rotation, as well as combined forms with translational changes like protraction [6]. Scapulothoracic orientation has been shown to be an important factor influencing simulated range of motion (ROM) in RTSA [2]. A CT-based study showed that with increasing thoracic kyphosis the scapula protracts, internally rotates and anteriorly tilts relative to the body axes [7]. Based on these findings, a classification of shoulder arthroplasty patients into different posture types has been suggested. Patients are categorized from Type A with upright posture and retracted scapulae over average Type B to Type C with kyphotic posture with protracted and internally rotated scapulae [7] (Fig. 1). Moroder et al. further investigated the impact of those static scapula orientation changes on ROM in RTSA, using a modified arthroplasty planning software that accounts for scapulothoracic orientation [2]. In RTSA the humeral component rotates in semicircular movements around the glenosphere. Since scapular internal rotation (SIR) dictates the orientation of the latter implanted glenosphere it was found to play a major role affecting simulated ROM, especially rotational movement [2, 7].

**Fig. 2:**
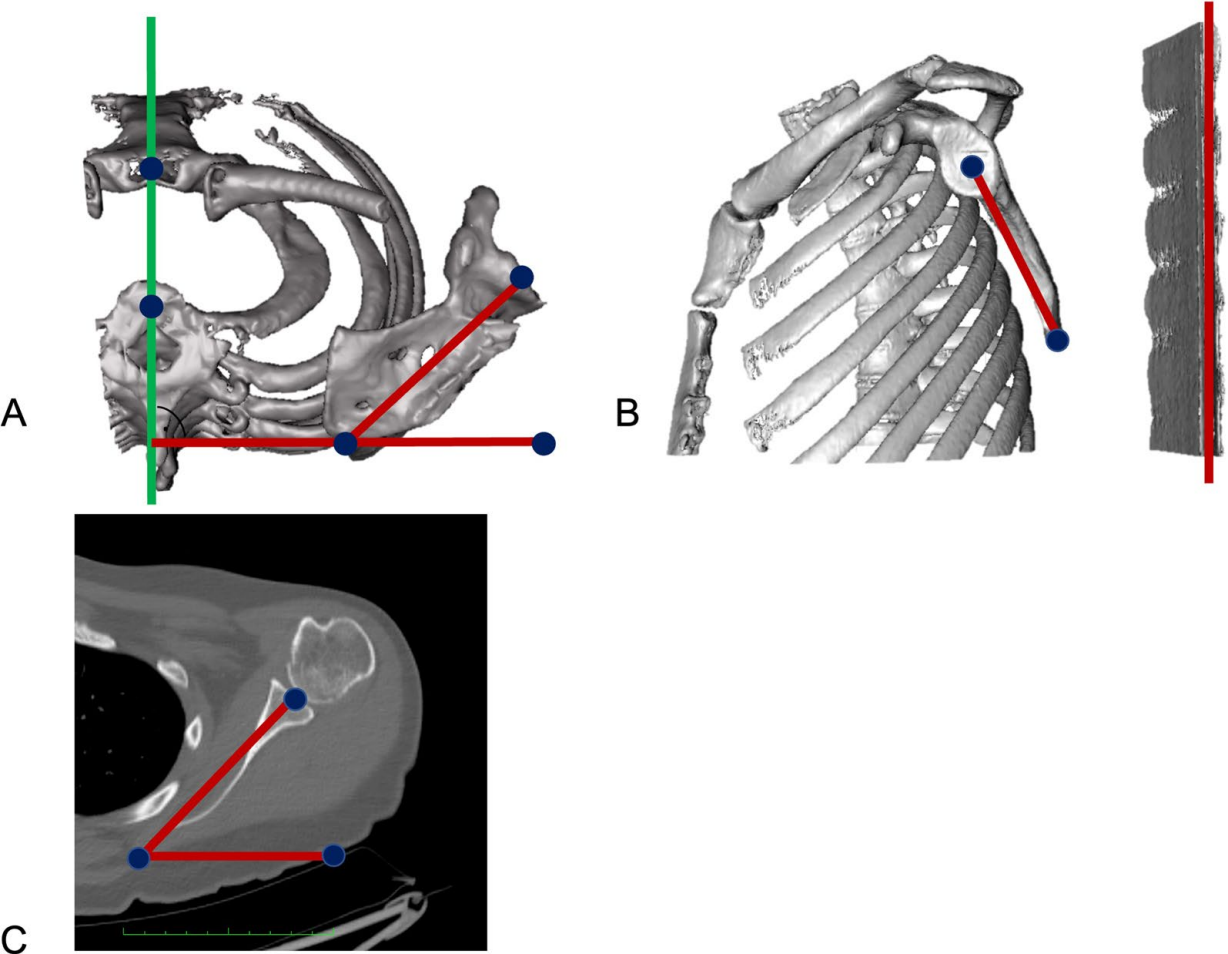
**A** Measurement of scapular internal rotation (SIR) in a 3D model in respect to the sagittal body axis. **B** Anterior scapular tilt was measured on an “en face” view of the glenoid in respect to the examination table as a reference of the coronar body axis. **C** 2D measurement of SIR in respect to the standardized transversal reference line with a limited field of view

Although geometrical considerations of these studies seem valid, measurements were conducted based on 3D-CT models of the entire torso, which is neither practical nor feasible due to radiation exposure. In a clinical setting surgeons rely on plain-radiographic imaging, clinical examination and possibly CT scans or MRI of the affected shoulder. For the determination of SIR, two obstacles need to be faced: First, the field of view (FOV) is often limited, and the spine not sufficiently depicted in the CT/MRI-scans, and therefore body axes cannot easily be determined. Furthermore, obtained values for scapulothoracic orientation might be affected by the supine position, as pressure to the medial ridge of the scapula might decrease internal rotation and protraction of the shoulder. Secondly, SIR is defined by the relation of the scapular axis (line from the trigonum scapulae to the center of the glenoid) to the transversal body axis. (Fig. 2) While clinically the trigonum scapulae can be accessed non-invasively through palpation, the center of the glenoid cannot be determined. Therefore, apical landmarks like the acromion, the coracoid process tip or the acromioclavicular (AC) joint are used to determine scapulothoracic orientation [6, 8–12]. Considering, that with increasing thoracic kyphosis the scapula shifts anteriorly, subsequent anterior tilt around the scapular axis is seen which could possibly alter the measured SIR based on apical landmarks.

Therefore, the two main objectives of this study are 1) to determine the difference between measurements of SIR in 2D CT scans without depicted spine and standard 3D measurements. And 2) to determine if clinical measurement of SIR based on apical scapula landmarks could be a valuable alternative. We hypothesize that measurement of SIR on 2D CT scans without depiction of the spine is reliable. We further hypothesize that the measurement of SIR in respect to the body axes is overestimated with progressive scapular tilt, regardless of the chosen apical landmark.

## Methods

We performed a search of our institutional database for patients who had received a whole-body positron emission tomography (PET)–computed tomography (CT) for non-shoulder-related indications (e.g., malignancies, inflammatory diseases) backdated from January 2020 until we had identified 50 patients who met the following inclusion criteria: (1) age 18 years or older; (2) supine positioning of the patient with both arms at the side and elbows resting on the examination table; (3) complete depiction of the trunk from the base of the skull to the pelvis; and (4) sufficient CT quality for three-dimensional rendering. Patients with visual pathologies of the upper extremities or thorax that potentially could alter scapular orientation, or scapulothoracic dimensions (e.g., fractures, prostheses, or dysplasia) were excluded. For each patient, both shoulders were analyzed as individual cases, which led to a total of 100 shoulders. CT imaging was performed with identical scan parameters and a primary slice thickness of 1.25 mm using a single scanner (Discovery MI; GE Healthcare, Chalfont St Giles, UK). The resulting study cohort consisted of 32 male and 18 female patients with a mean age of 61.2 ± 20.1 years (range 18; 91).

### Measurement of scapula orientation

Whole-body CT scans were exported, anonymized, and rendered into 3D models using Horos software (Horosproject.org; Nimble Co LLC d/b/a Purview in Annapolis, MD USA). As previously described [7], SIR was defined as an angle between a perpendicular line to the best-fit sagittal axis (midpoint from the vertebra body Th1 and midpoint of the sternum) and a line from the medial root of the scapular spine (trigonum scapulae) to the center of the glenoid (Fig. 2A). Anterior scapular tilt was defined as an angle between the midpoint of the glenoid and the inferior angulus and a line drawn vertically along the fixed examination table (Fig. 2B) on a parasagittal view in a 3D model. For independent determination of 2D scapular internal rotation, whole-body CT scans were cropped on an axial view leaving only the scapula and humerus from the acromion to the level of the inferior angulus and excluding visualization of the spine, comparable to a standard clinical CT scan field of view. Internal scapular rotation was then measured at the level of the deepest glenoid concavity as the angle between the midpoint of the glenoid to the medial root of the scapular spine and the standardized CT reference line for the transversal axis. (Fig. 2C).

Each shoulder (*n* = 100) was categorized into three different posture types (Fig. 1) for further subgroup analysis (Type A—upright posture, retracted scapulae; Type B—intermediate; Type C—kyphotic posture with protracted scapulae), based on the 3D measured SIR, as previously described [7]. The following threshold values were used: Type A ≤ 36°, Type B > 36° to 46°, and Type C ≥ 47° [2, 7]. Since each shoulder was evaluated separately, a patient could be categorized as two different types (e.g., 46° left would be Type B and 47° right would be Type C).

### Influence of anterior tilt on measurement of scapular internal rotation

**Fig. 3:**
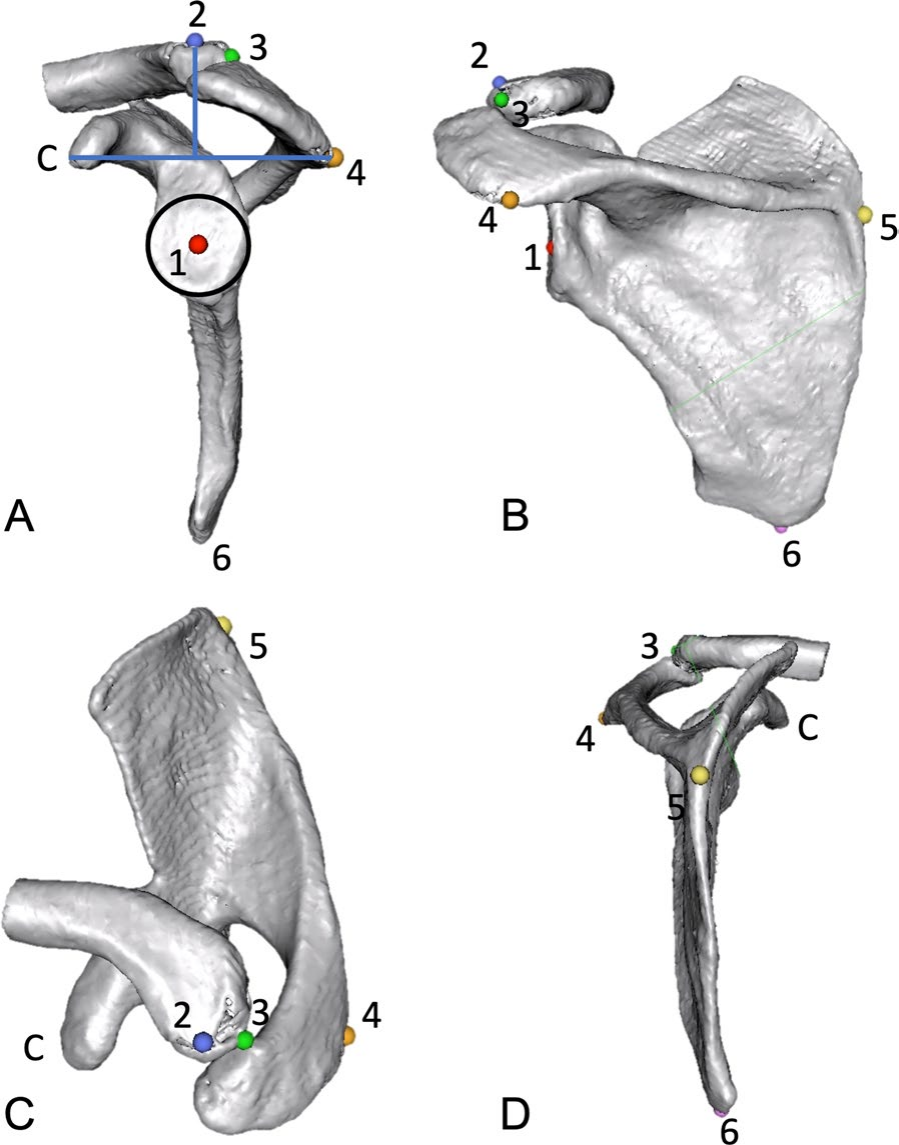
Various landmarks on the **A** parasagittal, **B** posterior-lateral **C** axial and **D** posterior view. 1: center of the glenoid; 2: most apical point located on a perpendicular line from the midpoint between the coracoid process and the angulus acromii; 3: posterior AC-joint; 4: angulus acromii, 5: medial root of scapular spine (trigonum scapulae); 6: inferior angulus; C: coracoid process tip

**Fig. 4:**
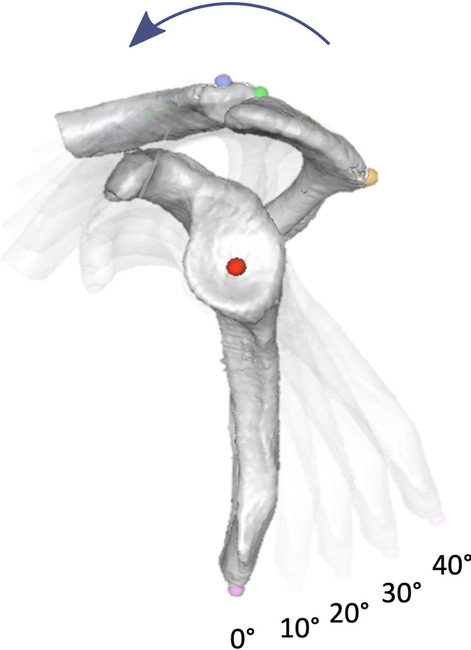
Parasagittal view with 0°, 10°, 20°, 30° and 40° anterior scapular tilt

**Fig. 5:**
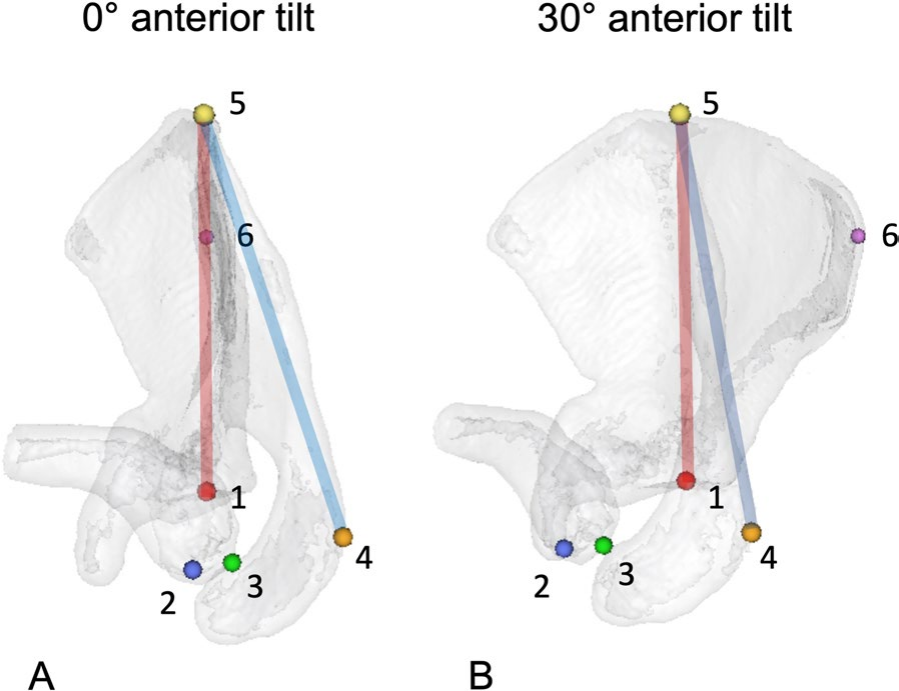
Examples of axial view of the scapula with **A** 0° and **B** 30° anterior tilt. Scapular axis is marked with a red line. An angle was measured from the glenoid center to the trigonum scapulae and to the respective landmark (blue line). 1: midpoint of the glenoid; 2: most apical point located on a perpendicular line from the midpoint between the coracoid process and the angulus acromii; 3: posterior AC-joint; 4: angulus acromii, 5: medial root of scapular spine; 6: inferior angulus

To determine the influence of anterior scapular tilt on the clinical measurement of SIR through palpable bony landmarks, 3D models of each scapula were created, removing the thorax and humerus. First, the following landmarks were set to define the scapular plane: center of the glenoid (midpoint of a best-fit circle on an en-face view), medial root of scapular spine (trigonum scapulae) and the inferior angulus. (Fig. 3A–D) Then bony landmarks were set, which could be used in a clinical examination: (1) the angulus acromia [13] (AA), (2) the most apical point of the posterior acromioclavicular (AC)- joint on the clavicular side [14], (3) and the most apical point located on a perpendicular line from the midpoint between the coracoid process tip (C) and the angulus acromii [15]. To determine the scapular axis, the marks of the glenoid and the medial root of the scapular spine are aligned on a parasagittal view and the inferior angulus is set vertically with 0° angle to mimic a neutral state. The scapula is then tilted anteriorly around the scapular axis in 10°, 20°, 30° and 40° (Fig. 4). On a transverse plane, a line from the glenoid center to the trigonum scapulae is drawn and the angles to the AA, AC-joint and midpoint between AA and C are measured. This is repeated for each step of scapular tilt (Fig. 5). Measurements were performed by the first author.

### Statistical analysis

For statistical analyses and descriptive data, we used SPSS Statistics Version 24.0 (IBM) software. All outcome variables were tested for normal distribution using the Kolmogorov–Smirnov test and showed a normal distribution. Intraclass correlation coefficients (ICC) with 95% confidence interval were calculated and interpreted according to Landis et al. [16] An ICC of 0.20 or less indicates slight agreement; 0.21 to 0.40, fair agreement; 0.41 to 0.60, moderate agreement; 0.61 to 0.80, substantial agreement; and 0.81 or greater, almost perfect agreement. Values from the first rater were used for further analysis. To compare 3D and 2D measurements of SIR an independent t-test was used. Furthermore, a linear regression was calculated, and a Bland–Altman-Plot created. Correlations between parameters (SIR, scapular tilt) were analyzed using the Pearson correlation coefficient (*R*). Sex differences were calculated by means of the independent t-test; differences between both shoulders, invariant analysis of variance. A *p*-value < 0.05 was considered significant.

## Results

### Intraclass correlation coefficients (ICC)

Measurements for 3D SIR were performed by the first author. In a prior study an almost perfect agreement was shown for these measurements between two raters with an intraclass correlation coefficients (ICC) of 0.87 for SIR and 0.89 for anterior scapular tilt [7]. Two orthopedic surgeons (P.S. and D.A.) conducted the measurements for 2D measurements of SIR independently with an almost perfect agreement (ICC = 0.97 with a confidence interval of 0.96; 0.98).

### Scapular internal rotation (SIR) and posture types

**Fig. 6:**
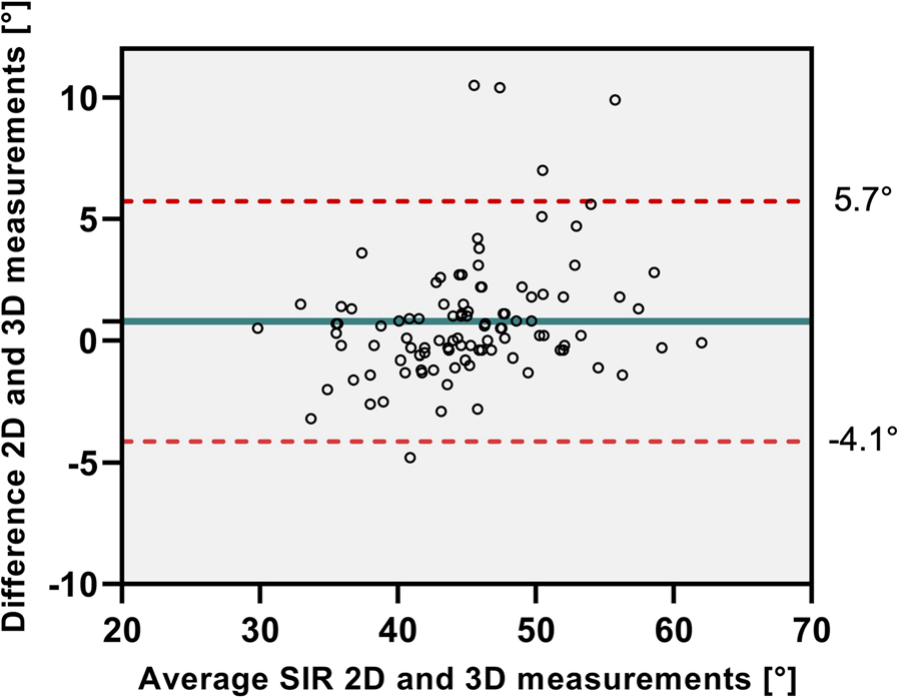
Bland–Altman-Plot showing differences between 3 and 2D measurements for scapular internal rotation (SIR). Mean difference is marked by blue line and upper and lower bound (± 1.96 SD) marked by dashed red lines

Mean SIR was 44.8° ± 5.9° and 45.6° ± 6.6° in the 3D and 2D model, respectively (*p* = 0.371). We categorized 11 shoulders as Type A, 55 Type B and 34 Type C based on measured 3D SIR. There was a linear regression between 3 and 2D measurements for SIR (*R* = 0.856; *p* < 0.001). Mean difference between the measurements was 0.8° ± 2.5° with a maximum of 10.5°. A Bland–Altman-Plot showed 5% outliers (± 1.96 SD) (Fig. 6). There was no significant difference between sexes for 3D SIR (*p* = 0.067) and 2D SIR (*p* = 0.094). Results for 2D and 3D measurements for SIR divided into posture types are summarized in Table 1. In a subanalysis all 5 shoulders (4 patients) who showed differences between 2 and 3D measurements outside the confidence interval were evaluated for thoracic scoliosis. A COBB angle was measured between the most tilted thoracic vertebrae above and below the apex of deformity. They showed a mean thoracic scoliosis of 18.8° ± 2.6° (range 11.1°; 17.2°). Two of those shoulders were categorized as Posture Type B and 3 as Type C.

**Table 1:** 2D and 3D measurement for SIR divided into posture types A, B and C

	2D SIR	3D SIR	*p*-value
Type A (*n* = 11)	35.1° ± 2.5°	34.7° ± 2.0°	0.671
Type B (*n* = 55)	43.5° ± 3.4°	42.8° ± 2.3°	0.328
Type C (*n* = 34)	52.4° ± 4.6°	50.8° ± 3.8°	0.188

*SIR* scapular internal rotation

*p* < 0.05*

*p* < 0.001**

### Scapular tilt

Mean anterior scapular tilt was 20.9° ± 5.0° (range 12.7; 39.5). There was a positive correlation between SIR and scapular tilt (correlation coefficient *R* = 0.471; *p* < 0.001). There was no significant difference between sexes for scapular tilt (*p* = 0.062). All results for scapular internal rotation and anterior tilt with respective subgroups (Type A, B and C) are summarized in Table 2.

**Table 2:** Measurement of scapulothoracic orientation divided into Posture Types A, B and C

Measurement	Type A	Type B	Type C	*p*-values		
	Mean ± SD	Mean ± SD	Mean ± SD	Type A vs. Type B	Type B vs. Type C	Type C vs. Type A
Scapular internal rotation 3D [°]	34.7° ± 2.0°	42.8° ± 2.3°	50.8° ± 3.8°	< 0.001**	< 0.001**	< 0.001**
Anterior scapular tilt [°]	16.1° ± 2.4°	19.9° ± 3.7°	24.2° ± 5.5°	0.025*	< 0.001**	< 0.001**

*p* < 0.05*

*p* < 0.001**

*, **reflects on whether *p* is < 0.05 or < 0.001 as stated in description, distinguishing high significance

### Influence of anterior tilt on measurement of scapular internal rotation

**Fig. 7:**
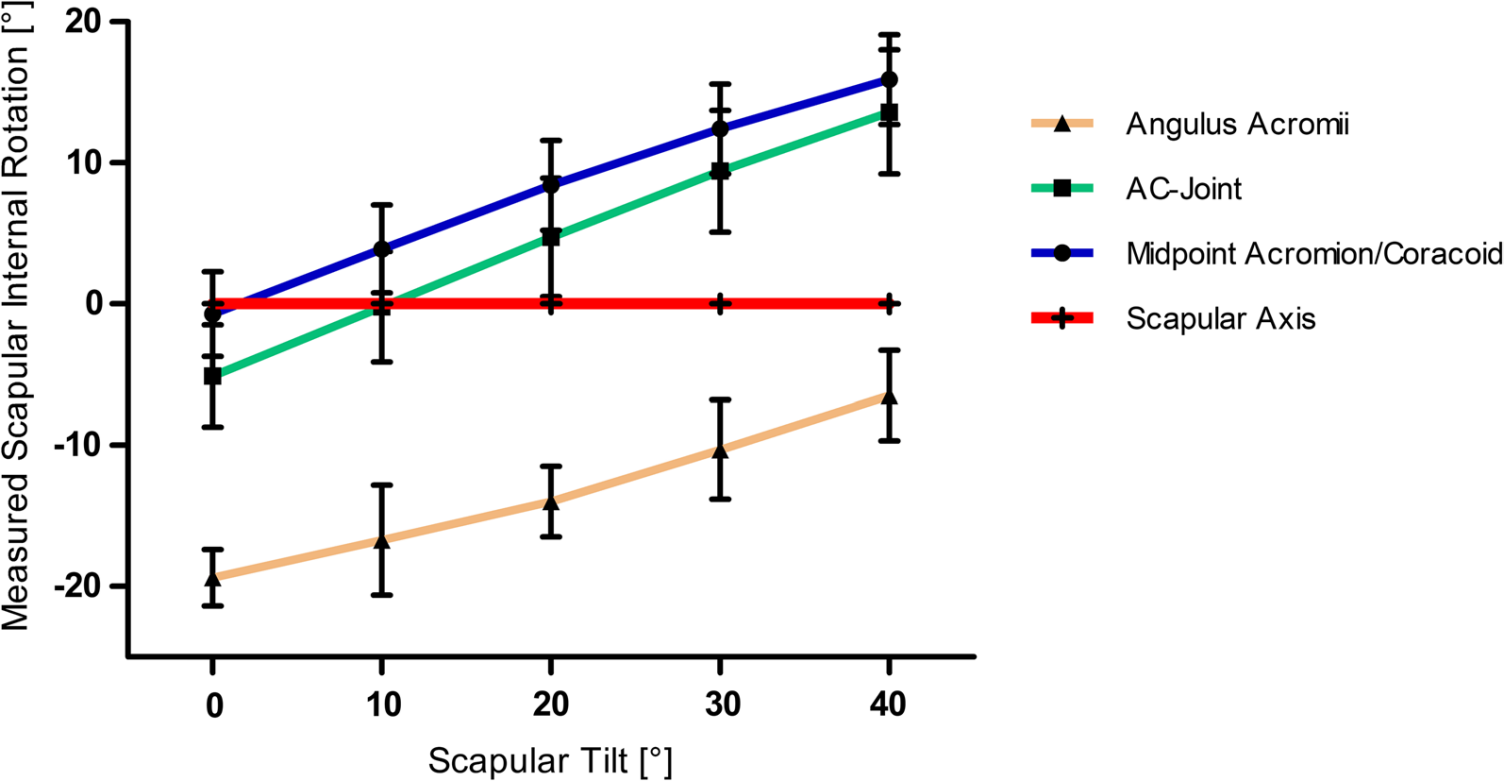
Results of measurement of scapular internal rotation (SIR) with SD based on different landmarks (Angulus acromii, AC- joint and midpoint AA/C) dependent on progressive anterior scapular tilt in 0°, 10°, 20°, 30° and 40°. Scapular axis is shown as a red line

Results for measurement of scapular internal rotation based on different landmarks (AA, AC- joint and midpoint AA/C) dependent on progressive anterior scapular tilt in 0°, 10°, 20°, 30° and 40° are shown in Fig. 7. Midpoint AA/C showed no significant difference to the scapular axis a 0° (*p* = 0.203) and the AC-joint at 10° anterior tilt (*p* = 0.949). All other point showed a significant difference from the scapular axis at all degrees of tilt. (Table 3) There were no sex related differences for scapular tilt measurements with the landmarks of the AA (*p* = 0.085) and midpoint AA/C (*p* = 0.054), but significant differences for the AC-joint (*p* < 0.001). We found no differences between both shoulders for any of the landmarks: AA (*p* = 0.774); midpoint AA/C (*p* = 0.138) and AC-joint (*p* = 0.752).

**Table 3:** Measurement of scapular internal rotation based on different landmarks with progressive anterior scapular tilt

Scapular tilt	0°		10°		20°		30°		40°	
	Mean ± SD	*p*-values	Mean ± SD	*p*-values	Mean ± SD	*p*-values	Mean ± SD	*p*-values	Mean ± SD	*p*-values
Angulus Acromii [°]	− 19.4 ± 2.0	< 0.001**	− 16.9 ± 2,8	< 0.001**	− 14.0 ± 2.5	< 0.001**	− 10.5 ± 2.8	< 0.001**	− 6.5 ± 3.2	< 0.001**
AC-joint [°]	− 5.1 ± 3.6	< 0.001**	− 0.2 ± 3.8	0.949	4.7 ± 4.1	< 0.001**	9.3 ± 4.3	< 0.001**	13.5 ± 4.4	< 0.001**
Midpoint AA/C [°]	− 0.7 ± 3.0	0.203	3.9 ± 3.1	< 0.001**	8.4 ± 3.2	< 0.001**	12.4 ± 3.2	< 0.001**	15.9 ± 3.2	< 0.001**

*p* < 0.05*

*p* < 0.001**

*, **reflects on whether *p* is < 0.05 or < 0.001 as stated in description, distinguishing high significance

## Discussion

Scapular internal rotation (SIR) has been recognized an important factor affecting shoulder movement in RTSA [1, 2, 7]. This study showed that even with a limited field of view in clinical CT scans, SIR can be reliably measured. However, values of 2D and 3D measurements need to be carefully considered due to supine position of the patient upon examination, which could diverge from scapulothoracic orientation in the standing or sitting position. When using apical superficial landmarks to determine SIR in the upright standing position we showed that anterior scapular tilt needs to be considered, as progressive changes alter the measurements.

Rotational movement of the arm, following RTSA, is subjected to changes of scapular orientation in the transverse plane, meaning that an increase in SIR favors internal arm rotation and vice versa [1, 2, 7]. In a clinical study, Sulkar et al. investigated patients following RTSA implantation with limited internal arm rotation in adduction [5]. Using biplane fluoroscopy they compared scapulothoracic orientation between patients with sufficient and poor rotational movement. They found that changes in scapula protraction and upward rotation influences internal arm rotation. Furthermore, dynamic changes in scapular tilt appeared to be one of the key factors to facilitate the achievable movement.

In a computer simulation study, body axes were used as a reference coordinate system to account for these changes [2]. It was shown that body posture influences the scapulothoracic orientation and therefore alters the simulated ROM. Patients with Posture Type C (poor posture) showed a lower simulated range of motion, regardless of component configuration. However, some disadvantages of poor posture might be counteracted with a modification of the prosthetic components by means of a lower neck-shaft angle and higher retrotorsion of the humeral component, as well a larger or inferior eccentric glenosphere [2]. With progressive internal rotation of the shoulder a mismatch between the opposition of the glenosphere and the humeral component is seen [7]. Although previous work was focused on RTSA, there might also be a relevance regarding preoperative planning in anatomic shoulder arthroplasty. The findings of this study should help the clinician to estimate these discrepancies, and further enhance an individualized approach.

**Fig. 8:**
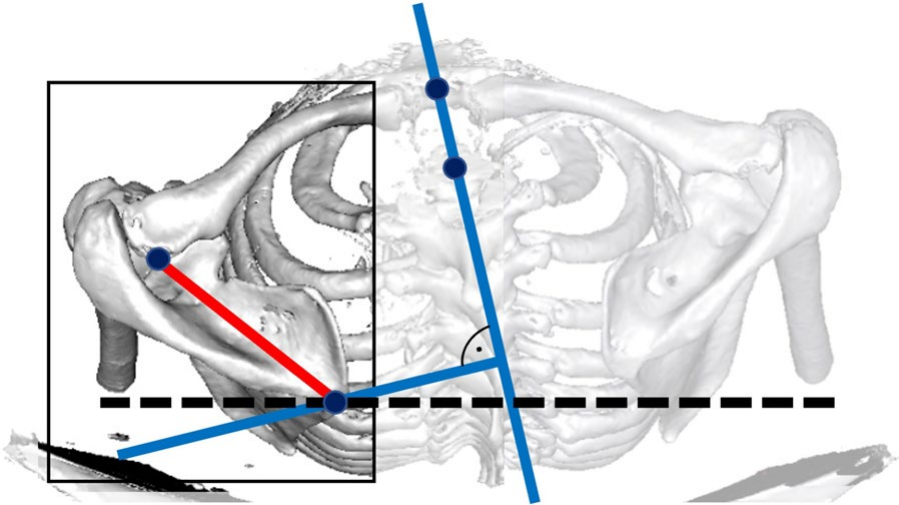
3D-CT reconstruction of a patient with advanced kyphoscoliosis. 3D measurement of scapular internal rotation (SIR) is referenced as a perpendicular line to the sagittal axis through vertebra Th1 and the sternum (blue line). With a limited field of view (black rectangle) the standardized CT reference (black dashed line) is used as the transversal axis

In this study it was shown that there was less than one degree mean difference in measured SIR between the established use of 3D whole-body CT scans, and 2D measurements with a limited FOV. However, the maximum difference between 2 and 3D measurement for SIR was 10.5°. While revaluating those outlier cases we noticed a difference between both shoulders especially in the 3D measurements. All patients were either categorized as Type B or C. Additionally we saw that there was a great discrepancy between the body axis marked by a line through the first thoracic vertebra and the sternum and the standard sagittal axis of the scanner. Therefore, we measured thoracic scoliosis in those patients and found mean values of 18.8°. It seems as if advanced thoracic scoliosis changes the alignment of the spine in a way that it potentially alters measured SIR for both sides (Fig. 8). This phenomenon though is only seen in the 3D measurements, as in 2D the reference line is determined by the position in the scanner. This case shows that the two techniques are not interchangeable in case of advanced scoliosis.

The determination of the scapular axis (Friedman line) on MRI for measurement of glenoid version or humeral head subluxation has been described before [17, 18]. Even though measurements in this study were conducted using CT scans, it can be assumed, that this method can be translated to MRI which typically also offers a limited FOV and comparable bony landmarks identification [19, 20].

While the transversal body axis can be easily determined clinically, the measurement of the scapular axis is limited, because the center of the glenoid cannot be assessed and therefore the bony landmarks used for CT- measurements cannot be utilized. However, surface-level landmarks can be used as substitutes to assess changes in scapulothoracic orientation and evaluate scapulohumeral rhythm [21, 22]. Due to skin shifting during movement invasive procedures like pin insertion into bony landmarks seem to be most accurate but certainly practically not feasible, which is why less-invasive procedures like motion sensors, biplane fluoroscopy and simple goniometer measurements are being considered [6, 8–11]. All in common, superficial scapular landmarks need to be defined [12]. A study by Ludewig et al. [10] investigated historical and current local scapular coordinate systems. The angulus acromii (AA) is a commonly used landmark to reference scapular orientation [12, 13]. Generally it is easily palpable even with thicker overlaying soft tissue and therefore suitable for detection of changes in scapular movement. However, our study shows that SIR is underestimated by around 19° when scapular tilt is at 0°. While they AA can be easily identified, the AC- joint and the midpoint between the coracoid process tip and the angulus acromii were described more reliable to match the center of the glenoid [14, 15]. The midpoint between the AA and C was anatomically the landmark closest to the center of the glenoid when scapular tilt was at 0°. By using this point, the trigonum scapulae and the inferior scapular angle, a scapular plane could be reconstructed. The AC-joint, on the other hand was closest to the scapular axis at around 10° of anterior tilt, with only about 5° variability between 10° and 20° anterior scapular tilt, which means that it would be suitable to measure SIR in Type A and B patients. However, in Type C patients with progressive anterior scapular tilt, scapular internal rotation would be highly overestimated with both landmarks but could still be underestimated with AA. In the literature often different landmarks for the local coordinate systems are being used, which makes the interpretation and comparison of the data difficult. A study by Kolz et al. [23] investigated if average rotation matrices can accurately convert kinematics between different local coordinate systems. The found that a conversion between systems using the posterior acromion, the AC-joint and the glenoid is possible within 4° root mean squared error.

Matsumura et al. investigated the difference between scapulothoracic orientation in supine and standing position using CT scans [24]. Interestingly, they found significantly less upward rotation, internal rotation and anterior tilt of the scapula in the standing position, although it was suspected that gravity led to an increase in internal rotation and anterior tilt. As described before, CT scans for analysis of SIR and anterior tilt were performed in the supine position, which could alter orientation and is one of the major limitations of this study. Nevertheless, this study underlines the need of an investigation of scapulothoracic orientation in the standing or sitting position.

Further limitations need to be noticed. As mentioned before, this study is an anatomic simulation study. For theoretical simulation of anterior scapular tilting the scapular axis was used as the rotational center, therefore, changes in upward/downward rotation were not considered, but might influence clinical measurements. Retrospective data of shoulder CT scans were used for the measurements without knowledge of shoulder related issues. However, for the purpose of this anatomical study we believe that the obtained measurements are comparable to arthroplasty patients, as positioning and scan parameters are equivalent. One major concern is that landmarks for the determination of SIR were set on 3D-CT models directly on the bone. Soft tissue was not considered, which could potentially alter the measurements. Additionally, patients with glenohumeral arthrosis were not excluded from this study, which might make the identification of landmarks in CT scans more difficult. Although not seen in our study cohort some patients might not be in a straight position on the examination table, which could alter the assumed body axes in the 2D measurement of SIR.

## Conclusion

2D CT scans are reliable to determine scapular internal rotation, even if the spine is not depicted, however, supine position is a limitation. When using clinical measurements with apical scapula landmarks, anterior tilt possibly alters measured SIR with progressive changes in scapulothoracic orientation (Posture Type C). In patients with low anterior scapular tilt (Posture Type A and B) the AC-joint is the landmark most reliable to match the scapular axis.

## Data Availability

The datasets used and analyzed during the current study are available from the corresponding author on reasonable request.

## Abbreviations

AA: Angulus acromii
AC: Acromioclavicular
C: Coracoid process tip
FOV: Field of view
ROM: Range of motion
RTSA: Revere total shoulder arthroplasty
SIR: Scapular internal rotation
